# Dancing in Shackles: The Double-Edged Sword Effect of Felt Accountability on Work Outcomes and Individual Wellbeing

**DOI:** 10.3389/fpsyg.2022.904946

**Published:** 2022-06-10

**Authors:** You Li, Wei Liu, Guangtao Yu

**Affiliations:** Department of Organization and Human Resource Management, Business School, Central University of Finance and Economics, Beijing, China

**Keywords:** felt accountability, obsessive passion, work overload, task performance, emotional exhaustion, self-determination theory

## Abstract

Accountability is a core element for groups and societies to operate efficiently. However, there have been confusing findings in previous studies on felt accountability, and few efforts have been made to clarify its complicated role. Drawing on self-determination theory, we developed an integrative model to examine the double-edged sword effect of felt accountability on work outcomes and individual wellbeing. We utilized a three-wave sample of 294 employees to test our hypotheses. The findings supported our hypotheses. Specifically, felt accountability is positively related to both task performance and emotional exhaustion, and obsessive passion mediates the positive relationship between felt accountability and task performance, while work overload mediates the positive relationship between felt accountability and emotional exhaustion. This study integrates the positive and dark sides of felt accountability, provides new insights into its mechanism and expands the application of self-determination theory.

## Introduction

*I acknowledge that an accountability system is necessary, and only regular supervision and accountability can ensure that duties are carried out. But it does put a tremendous amount of pressure on me… if I don’t do well, I will not only fail to meet the expectations of my leaders but also drag down my team.*

—An employee had said when asked how he thought about the accountability system in the company

Due to the high uncertainty of the economic environment brought about by COVID-19, enterprises are faced with unprecedented challenges of survival and development. Accountability is the key to improving employee execution, promoting internal coordination, and safeguarding organizational efficiency. As Ashkenas (2012) points out in the *Harvard Business Review*, one of the most sacred tenets of management is the need for clear accountability. In HR practice, accountability is one of the foundational systems widely implemented in organizations for functional division and organizational design, including work procedures, performance monitoring, and reward policies (Kuo et al., 2021). By requiring members to be accountable for the organization’s goals and explaining or justifying their actions to others (Mackey et al., 2018), accountability constrains the individuals’ work behavior as an instrument for managers in performance appraisal and sanctions (Overman and Schillemans, 2022). In contrast to the objective system, success or failure of enterprises is more influenced by how employees perceive their accountabilities (Park, 2018; Dewi and Riantoputra, 2019). Felt accountability is a subjective reflection of accountability and defined as perceived expectations that one’s actions or decisions will be evaluated by a specific audience, and rewards or punishments are conditioned on this evaluation (Hall et al., 2006). As an essential mechanism for explaining workplace phenomena, felt accountability significantly impacts individuals’ emotions, cognitions, behaviors, and decisions (Hall et al., 2017).

As scholars and managers call for attention to the construction of accountability at the individual level, research on felt accountability is increasing. However, there is still much unknown about this construct, thus requiring further investigation (Hall et al., 2017; Dewi and Riantoputra, 2019). Previous studies showed contradictory results on the effects of felt accountability. On the one hand, some studies have revealed the positive effects of felt accountability, such as task attentiveness (Frink and Ferris, 1998; Mero et al., 2006), complex decision-making strategies (Mero et al., 2014), and innovative work behavior (Kuo et al., 2021), which imply that felt accountability is a positive construct. On the other hand, another stream of studies viewed felt accountability as a stressor with negative effects, such as job tension (Hochwarter et al., 2005; Breaux et al., 2009), feelings of insecurity (Mackey et al., 2018), workplace conflict (Hall et al., 2017), and reduced job satisfaction (Lanivich et al., 2010). The contradictory results imply that felt accountability as a complex phenomenon is not black or white, but a double-edged sword for individuals in the organization (Hall, 2005). As Hall et al. (2017) mentioned that there might be different effects of felt accountability on job outcomes and individual wellbeing. However, there is a lack of empirical evidence to support this view. Few efforts have been made to clarify the complicated role of felt accountability. Given this research gap, this study focuses specifically on the following questions: Whether and How does felt accountability have disparate impacts on work outcomes and individual wellbeing, and what is its mediating mechanism?

Almost all studies have relied on conservation-of-resources theory and role theory, but neither is convincing and thorough in explaining the mechanism of felt accountability. First, conservation-of-resources theory overemphasizes the negative effects of felt accountability as a resource threat, ignoring the fact that it not only causes stress but also brings about work effort. Second, although role theory considers role expectations from others as an essential component of felt accountability (Frink and Klimoski, 2004) and explains the mechanism based on this. Due to the lack of consideration of motivations for individual behavior, role theory cannot clearly explain why individuals react differently to role expectations and why individuals take actions consistent with external role expectations (Biddle, 1986). Therefore, to open the black box of the mechanism of the felt accountability, it is necessary to introduce the perspective of motivation.

From the perspective of internalization and motivation, self-determination theory can better explain the nature of felt accountability and provide an insight into the underlying mechanisms. First, self-determination theory is an organismic theory of human behavior and personality development, suggesting that individuals tend to internalize important external norms, expectations, and values (Ryan and Deci, 2017). Based on differences in the degree of internalization and integration, self-determination theory distinguishes between two types of individual motivation: autonomous motivation and controlled motivation. The latter refers to the motivation to engage in activities with a sense of being forced by external conditions or internal psychology (Ryan and Deci, 2000a). Consistent with self-determination theory, felt accountability is a typical organic integration of the individual with the external norms (objective accountability in organizations) and is associated with the controlled internalization process. Second, self-determination theory provides a better explanation for the mechanism of felt accountability. Obsessive passion is a strong tendency for individuals to engage in an activity beyond their control, deriving from the controlled internalization process (Vallerand et al., 2010). Then, felt accountability, which can be view as a typical controlling factor (e.g., external audience expectations and evaluations, organizational rewards, and punishments) (Dewi and Riantoputra, 2019), enhances obsessive passion by facilitating controlled internalization processes. By reducing the individual’s experience of autonomy, felt accountability leads to work overload, and thus brings about emotional exhaustion (Ryan and Deci, 2017).

Therefore, drawing on self-determination theory, we developed an integrative model to examine the double-edged sword effect of felt accountability. In the theoretical model, we expect that felt accountability is positively related to task performance and emotional exhaustion. Specifically, felt accountability promotes task performance *via* obsessive passion, but induces emotional exhaustion *via* work overload at the same time.

We tested our research model utilizing a three-wave sample of 294 employees. The findings generally supported our hypotheses. This study contributes to the literature in three ways. First, by empirically examining the double-edged sword effect, this study contributes to the research on felt accountability in organizational management. Almost all studies examined the role of felt accountability from a single path, failing to take a comprehensive view of its positive and dark sides. We place work outcomes and individual wellbeing within a model for the first time, distinguish the results of felt accountability on different targets, and thus dialectically view the benefits and costs it brings about. Second, this study expands the theoretical perspective of felt accountability and provides new insights into its mechanism. Previous studies are based on role theory or resource conservation theory, but neither of these perspectives provides a comprehensive explanation. Drawing on self-determination theory, we examine the mediating roles of obsessive passion and work overload, and reveal different pathways of felt accountability on task performance and emotional exhaustion. Third, for self-determination theory, previous studies have focused more on autonomous motivation, neglecting the use and exploration of the controlled motivation. By extending the controlled motivation perspective of self-determination theory into the area of felt accountability, this study expands the application of self-determination theory. Also, this study has practical implications for organizational management. By revealing the double-edged sword effect of felt accountability, we provide deep insights for managers to make proper use of accountability mechanisms, to protect employees’ psychological wellbeing and achieve sustainable development.

## Theory and Hypothesis

Self-determination theory (Deci and Ryan, 1985) is an organismic theory of human behavior and personality development, focusing on the theme of how social contextual factors support or hinder an individual’s development by satisfying their basic psychological needs (Deci et al., 1989; Ryan and Deci, 2000b). The basic assumption of self-determination theory is that humans are naturally curious, physically active, and highly social individuals who tend to absorb, assimilate and integrate the values and norms of the external environment. This tendency leads to internalization, which refers to the internal psychological process of absorbing values, expectations, or behavioral norms from the organizational environment and transforming them into their own (Ryan and Deci, 2017). Self-determination theory suggests that the process of internalization can be characterized on a continuum, from controlled to relatively autonomous (Ryan and Deci, 2017). Based on differences in the degree of internalization and integration, this theory distinguishes between two types of individual motivation: autonomous motivation and controlled motivation. Specifically, autonomous motivation refers to engage in activities with a sufficient sense of willingness, will, and choice. In contrast, controlled motivation is the instrumental motivation to engage in activities with a sense of being forced or controlled by external conditions or internal psychology, for example, to obtain external rewards and social recognition, or to avoid external penalties (Ryan and Deci, 2000a).

The core idea of self-determination theory holds that individuals have three basic psychological needs, including competence, relatedness, and autonomy. Autonomy is the basic need for individuals to experience a sense of psychological freedom to engage in activities based on their own will and choices. Competence is the basic need for individuals to influence the environment and feel develop their abilities directly. Relationships is the basic need for individuals to experience a connection to society. Satisfaction of the three needs is critical for work performance and wellbeing, and failure to meet any of these needs would lead to adverse consequences (Deci et al., 1989). When basic psychological needs are blocked, individuals are more focused on external outcomes and promote controlled motivation, which negatively affects behavioral outcomes and health (Gagne and Deci, 2005). Thus, controlled motivation may motivate individuals to exert effort to obtain instrumental purposes, such as specific task performance (Zhang et al., 2020), but is unlikely to improve health and wellbeing.

In the context of this research, we consider felt accountability as a controlled motivation associated with controlled processes. According to the literature, a motivator is likely to be controlled if it satisfies the following characteristics: (a) personal obligation; (b) perform under pressure; (c) behavior required for instrumental purposes (Deci and Ryan, 1985). Felt accountability is consistent with these characteristics. First of all, felt accountability emphasizes the obligation to take responsibility for the work results. Second, due to the emphasizing of the reward or punishment brought about by the evaluations (Hall et al., 2006), felt accountability leads to pressure and makes individuals comply with work requirements (e.g., performance requirements, workflow) for fear of mistakes (Frink and Ferris, 1998). Finally, individuals with high felt accountability tend to meet external expectations and maintain their self-esteem, image, or status (Kuo et al., 2021). Therefore, they are more likely to be motivated by external instrumental purposes, producing controlled internalization processes (Zhang et al., 2020).

Therefore, based on self-determination theory, we propose an integrative model that suggests felt is likely to be experienced as a controlled motivation associated with controlled processes, and has different impacts on work outcomes and individual wellbeing. We argue that felt accountability promotes task performance through obsessive passion, but increases emotional exhaustion through work overload.

### Felt Accountability, Task Performance, and Emotional Exhaustion

Accountability is a system of rewards and punishments designed to ensure that individual behavior is in line with organizational standards (Frink and Ferris, 1998). Felt accountability is defined as perceived expectations that one’s actions or decisions will be evaluated by a specific audience, and rewards or punishments are conditioned on this evaluation (Hall et al., 2006). Compared to accountability as an external public process, felt accountability is considered to be an internal process that emphasizes the individual perception of norms (Cummings and Anton, 1990). Previous studies indicate that felt accountability significantly impacts emotions, cognition, behavior, and decision making (Hall et al., 2017). Task performance is one of the most significant work behaviors in the workplace, which refers to meeting formal job requirements and reflects performance indicators that directly evaluate work results, such as the tasks and responsibilities specified in a job description. It involves non-discretionary, in-role behavior that focuses on efficiency (Yu and Frenkel, 2013).

We argue that felt accountability positively predicts task performance. First, felt accountability is associated with maintaining interpersonal relationships (Frink and Ferris, 1998; Overman and Schillemans, 2022). Self-determination theory proposes that to satisfy the need to belong and gain the acceptance and recognition of the organization’s members, they tend to internalize and obey important external norms, thus are willing to contribute to the organization (Ryan and Deci, 2017). Individuals with high felt accountability tend to explain their behavior in response to external evaluations (Mackey et al., 2018) and actively maintain interpersonal relationships to gain a sense of belonging as they seek acceptance and approval from others (Kuo et al., 2021). As a result, they are willing to adhere to strict performance standards, put effort into their work, and contribute to others.

Second, since felt accountability reflects the extent to which individuals take responsibility for work results (Hall et al., 2017), those with high felt accountability pay more attention to the results. Therefore, to ensure that work behaviors and decisions are appropriate, they put a high level of cognitive effort into analyzing and gathering information related to their job responsibilities (Kuo et al., 2021). Previous studies also found that high accountability has positive effects on task attentiveness (Mero et al., 2006), job competency (Laird et al., 2009), and performance (Wallace et al., 2011). Integrating the above arguments, we hypothesize the following:

*Hypothesis H1a: Felt accountability is positively related to task performance.*

Thus far, we have proposed that felt accountability motivates individuals to conform to these norms, and has a positive impact on work outcomes (task performance). However, scholars point out that felt accountability not only brings about work effort but also causes stress (Mero et al., 2014). As the main component of job burnout (Maslach and Jackson, 1981), emotional exhaustion is a chronic state of emotional and physical exertion. It manifests as feeling overstressed, depleted of energy, physical and mental exhaustion, and loss of energy (Maslach and Schaufeli, 2001). As a stress response to work, emotional exhaustion could lead to work-related depression and physical illness (Cropanzano et al., 2003). We propose that felt accountability positively affects employee emotional exhaustion. Then, we turn our attention to the perspective of motivation and analyze this relationship.

According to self-determination theory, among the three basic psychological needs, autonomy is particularly important and facilitates the satisfaction of the other two needs, whereas controlling environments or events can disrupt the fulfillment of autonomy (Ryan and Deci, 2017). In essence, as a controlling factor, felt accountability (Frink and Ferris, 1998) trigger emotional exhaustion by disrupting the need for autonomy. First, individuals with higher felt accountability may be more tentative, cautious in actions, and likely to exert more emotional effort in response to expectations and trust (Hall et al., 2017), leading to irritability and pain. Second, felt accountability emphasizes rewards or punishments resulting in the evaluation. Individuals with high felt accountability are more aware of the seriousness of external norms and the fear of making errors. Therefore, this will increase psychological bondage and depression, even making employees feel that work and life are a continuous, numbing endurance (Hochwarter et al., 2005; Hall et al., 2006; Lanivich et al., 2010). Given all of that, we propose Hypothesis 1b:

*Hypothesis H1b: Felt accountability is positively related to emotional exhaustion.*

### From Felt Accountability to Task Performance: The Mediating Effect of Obsessive Passion

Work passion is a strong tendency of organizational members toward preferred activities that individuals enjoy, value, and to which they devote their time and energy (Vallerand et al., 2003). Stemming from self-determination theory, Vallerand et al. (2003) initially advanced the dualistic model of passion. This model proposed that passion can be classified into two forms, harmonious passion, and obsessive passion. Harmonious passion is defined as a strong desire of an individual to voluntarily participate in a favorite work, freely choosing to engage in an activity and recognizing its significant value. Obsessive passion is a strong tendency for individuals to engage in an activity beyond their control. Scholars have proposed that whether individuals develop harmonious or obsessive passions depends on different internalization processes brought about by social and personal factors (Séguin-Levesque et al., 2003; Vallerand et al., 2010). That means, harmonious passion is generated by the autonomous internalization process, and is not influenced by additional pressures outside the activity. In contrast, obsessive passion is generated by the controlled internalization processe, which is associated with a utilitarian purpose attached to the activity, such as interpersonal pressure, social evaluation, and performance requirements (Perttula and Cardon, 2011). Individuals with obsessive passions are not free to make choices, feeling controlled and compelled to engage in work activities (Pollack et al., 2020).

We suggest that obsessive passion, as an important outcome of controlled internalization processes (Vallerand et al., 2010), is a critical factor in linking felt accountability and task performance. In one aspect, due to the association with controlling factors, felt accountability promotes controlled internalization processes, positively affecting obsessive passion. According to self-determination theory, organic integration inclines individuals to internalize important expectations endorsed by significant others, and the controlled internalization process is associated with two aspects, including external control and internal control. Specifically, external control is derived from extrinsic stimuli, such as rewards and evaluation. Internal control is derived from self-factors involving episodic self-esteem, self-involvement, and self-monitoring (Ryan and Deci, 2017). First, in terms of external control, given that external evaluations are increasingly dominant, individuals with high felt accountability are compelled to perform their jobs, generating the typically controlled internalization process, thus resulting in obsessive passion (Bélanger et al., 2015). Second, in terms of internal control, previous research suggests that self-esteem contingencies and self-awareness maintenance are antecedents to obsessive passion (Mageau et al., 2011). Individuals with high felt accountability are likely to overvalue their image and status, and engage in activities that enhance self-esteem and self-awareness, for example, maintaining self-images in public (Schlenker et al., 1994; Hall et al., 2017), and managing impressions (Hall, 2005; Breaux et al., 2009; Hall et al., 2017). Third, because it reflects the degree of personal responsibility for important work outcomes (Hall et al., 2006), felt accountability implies that individuals need to fulfill their obligations to others and devote significant time and energy to work, which serves as an influential factor in the formulation of obsessive passion. To sum up these arguments, we hypothesize the following:

*Hypothesis 2a: Felt accountability is positively related to obsessive passion.*

Furthermore, we argue that felt accountability enhances task performance by stimulating obsessive passion. First, obsessive passion leads to more work engagement. Because it is difficult to control themselves from not working, individuals with high levels of obsessive passion exhibit a rigid persistence (Vallerand et al., 2003) by devoting more time and energy to their work (Pollack et al., 2020). Second, obsessive passion leads to greater dedication to work. Since work occupies a significant proportion of personal identity (Vallerand et al., 2003; Lalande et al., 2017), individuals with high levels of obsessive passion place a high priority on work as an essential way to achieve self-worth (Ryan and Deci, 2017), thus willing to sacrifice and dedicate to work. Given the same external environment, individual task performance depends on work dedication and effort (Zhang et al., 2020). Therefore, we propose the following:

*Hypothesis 2b: Obsessive passion mediates the positive relationship between felt accountability and task performance.*

### From Felt Accountability to Emotional Exhaustion: The Mediating Effect of Work Overload

Work overload refers to the task-based stress that occurs when individuals fail to fulfill organizational commitments, responsibilities, and job requirements, due to insufficient time and energy (Peterson et al., 1995). Employees’ work overload is related to two aspects, quantity and quality of work. We explain the impact of felt accountability on work overload by analyzing these two factors. First, as for quality, according to the cognitive evaluation perspective in self-determination theory, the evaluations based on the threat of reward or punishment have a clear functional significance of control, reducing the experience of autonomy and undermining internal motivation, thus leading to stress and annoyance (Ryan and Deci, 2017). Felt accountability arises from rewards, punishments (Mackey et al., 2018), and evaluations that imply more demanding work and a higher quality of work (Kuo et al., 2021). Individuals with high felt accountability are more inclined to feel threatened by these additional requirements. To reduce this concern, individuals are required to put in sustained effort, sacrifice personal time off, or are even forced to deviate from their normal lives, thus triggering role stress.

Second, as for quantity, according to role theory, roles in organizations arise from normative expectations that reflect formal demands from the organization and pressures from informal groups. When individuals have difficulty meeting two or more expectations, role strain arises (Kahn et al., 1964; Biddle, 1986). Consistent with arguments, felt accountability is generated by the expectations of various “audiences” (e.g., superiors, colleagues, and even individuals themselves) in the organization (Hall et al., 2017), which do not directly increase workload but extend the sense of responsibility and scope of the work role (Cheong et al., 2016). This process will interfere with the role that was constructed previously, which in turn leads to an increase in role stress. Thus, we propose the following:

*Hypothesis 3a: Felt accountability is positively related to work overload.*

Moreover, we argue that work overload, as a typical job stressor, positively affects emotional exhaustion. On the one hand, in order to cope with heavy work, individuals need to make more work investment and pay more emotional labor. However, due to the lack of adequate rest and entertainment, individuals find it difficult to recover from exhaustion and thus feel depressed at work. On the other hand, individuals with high work overload are constantly threatened with losing their jobs because of the difficulty in meeting the expectations and trust of others (Cheong et al., 2016). In other words, heavy workloads make individuals doubt and uncertain about their ability, which can lead to feelings of anxiety and worry (Acosta-Prado et al., 2020). Empirical results have shown that work overload is strongly associated with negative emotions and psychological problems (Lee and Ashforth, 1996; Spector and Jex, 1998), providing evidence for the above arguments. As noted above, we propose that:

*Hypothesis 3b: Work overload mediates the positive relationship between felt accountability and emotional exhaustion.*

**FIGURE 1:**
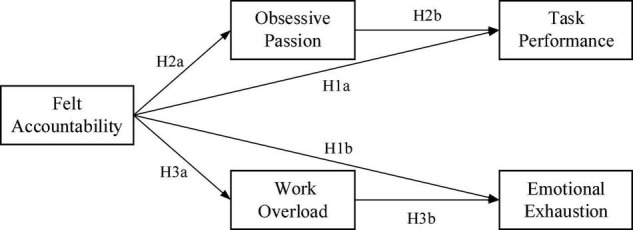
Research model.

In summary, we presented our conceptual framework in Figure 1.

## Method

### Data Collection Procedures

From April 2021 to June 2021, data were collected from employees and managers in various industries across China. Through a professional online data survey platform (Credamo), we compiled an online questionnaire and recruited participants. Before the survey, we set up distributions through this data survey platform. First, to ensure that samples fit the research context, we restricted the identity of the participants only to office workers (managers and general staff). Second, to improve the quality of the questionnaire, we selected participants with a previous adoption rate of at least 60%. In addition, we added screening questions and specified options in the questionnaire design to prevent participants from not reading the questions. At last, we informed participants that the survey was anonymous and voluntary, and the survey data was strictly confidential.

To avoid the potential for common method variance, we collected the data in three waves, separated by 2 weeks. In the first wave (*T1*), participants reported on felt accountability and provided their demographic information. In the second wave (*T2*), participants reported on obsessive passion and work overload. In the third wave (*T3*), participants reported on task performance and emotional exhaustion. Initially, we sent out 400 questionnaires, 382 valid questionnaires were collected, and we created a sample pool for these 382 participants. Two weeks later, for the 382 participants who had already participated in the survey at *T1*, we targeted the second wave questionnaire, and 342 valid questionnaires were collected. Another 2 weeks apart, we targeted the third wave questionnaire for the 342 employees who had already participated in the survey at *T1*, *T2*, and 321 valid questionnaires were collected. After matching responses in three waves, we obtained a sample of 321 participants. We eliminated 27 questionnaires that did not pass the screening questions and were filled in haphazardly. Finally, we obtained 294 valid questionnaires.

Of the final participants, the average age was 29.72 years, 53.1% were male, and the average work tenure was 3.06 years in their current organization. In terms of education, 2.4% had a high school education or below, 9.2% had a diploma degree, 81.0% had a bachelor’s degree, and 9.9% had a master’s degree. In terms of the unit properties, 21.1% were in state-owned enterprises, 53.4% were in private enterprises, 12.9% were in foreign-owned enterprises, 10.9% were in public institutions, and 1.7% were in other types of units.

### Measures

The scales we used were all published in international journals. We asked two bilingual Ph.D. students to follow a rigorous translate-back translation procedure of the English scales for the purpose of ensuring accuracy. Seven-point Likert scale was used in questionnaires (1 = strongly disagree and 7 = strongly agree).

#### Independent Variable: Felt Accountability

We measured felt accountability using eight items from Hochwarter et al. (2007) at Time 1. Sample items include I am held accountable for my actions at work, I often have to explain why I do certain things at work, and the jobs of many people at work depend on my success or failures (Cronbach’s α = 0.827).

#### Mediator 1: Obsessive Passion

We measured obsessive passion using seven items from Vallerand et al. (2003) at Time 2. This scale has two dimensions, harmonious passion and obsessive passion. Each dimension has seven measurement entries, and we selected obsessive passion. Sample items include I am emotionally dependent on this activity, I have a tough time controlling my need to do this activity, and The urge is so strong, I can’t help myself from doing this activity (Cronbach’s α = 0.850).

#### Mediator 2: Work Overload

Work overload was measured at Time 2 using the 5-item measure developed by Peterson et al. (1995). Sample items include I feel overburdened at work, I need to be relieved of some work, and My workload is so heavy that I can’t guarantee quality (Cronbach’s α = 0.942).

#### Dependent Variable 1: Emotional Exhaustion

Emotional exhaustion was measured at Time 3 using the 6-item measure developed by Maslach and Jackson (1981). Sample items are I feel emotionally drained from my work, I feel frustrated by my job, and Working with people all day is really a strain for me (Cronbach’s α = 0.926).

#### Dependent Variable 2: Task Performance

Task performance was measured at Time 3 using the 6-item measure developed by William and Anderson (1990). Sample items are Adequately completes assigned duties, Fulfills responsibilities specified in job description and Meets formal performance requirements of the job (Cronbach’s α = 0.861).

#### Control Variables

Previous studies have indicated that individual background variables, including gender, age, education, and work tenure, may be factors influencing employee task performance and emotional exhaustion. Therefore, we controlled for the effects of these variables. Moreover, we also control for the type of unit, an organizational-level variable, because the types of units in China may influence the results due to the differences in informal systems.

### Analytic Strategy

We used SPSS 25.0 and AMOS 24.0 to analyze the data in this study. First, we conducted descriptive statistics and correlation analysis with SPSS 22.0. Second, as suggested by Podsakoff et al. (2012), we tested for common method bias using Herman’s one-way test (Podsakoff et al., 2012). Third, since the model had multiple dependent variables, drawing on the experience of previous studies (Harju et al., 2021), we tested the hypothesis by structural equation modeling (SEM) (Marsh et al., 2004; Jackson et al., 2009). Specifically, we developed a structural equation model and conducted a mediating effects test using AMOS 24.0, in which felt accountability was the independent variable, obsessive passion and work overload were mediating variables, task performance and emotional exhaustion were dependent variables.

## Results

### Descriptive Statistics and Correlations

As shown in Table 1, we present means, standard deviations, and correlations between variables, which preliminarily support our hypotheses. Felt accountability was positively related to obsessive passion (*r* = 0.354, *p* < 0.01), task performance (*r* = 0.153, *p* < 0.01), work overload (*r* = 0.443, *p* < 0.01), and emotional exhaustion (*r* = 0.222, *p* < 0.01). Additionally, work overload was positively related to emotional exhaustion (*r* = 0.457, *p* < 0.01) and was negatively related to task performance. Interestingly, obsessive passion was positively related to task performance (*r* = 0.383, *p* < 0.01), but negatively related to emotional exhaustion (*r* = 0.080, ns).

**TABLE 1:** Means, standard deviations, and correlations.

Variables	*M*	SD	1	2	3	4	5	6	7	8	9	10
1. Gender	0.470	0.500	–									
2. Age	29.720	5.143	–0.037	–								
3. Education	2.980	0.512	0.005	–0.059	–							
4. Tenure	3.060	0.901	−0.166**	0.636**	–0.013	–						
5. Organization type	2.190	0.947	0.073	–0.019	0.063	−0.149*	–					
6. Felt accountability	4.319	1.023	0.020	0.154**	0.057	–0.016	0.016	–				
7. Obsessive passion	4.594	1.072	0.109	0.245**	0.074	0.191**	–0.050	0.354**	–			
8. Work overload	4.029	1.524	0.049	0.039	0.069	–0.059	0.077	0.443**	0.080	–		
9. Task performance	5.119	0.984	0.073	0.099	0.030	0.118*	–0.099	0.153**	0.383**	−0.249**	–	
10. Emotional exhaustion	3.299	1.373	–0.057	−0.134*	0.022	−0.185**	0.111	0.222**	−0.229**	0.457**	−0.456**	–

N = 294. Gender: 1 = Male; 2 = Female. Age and tenure are the actual number of years. Education: 1 = High school and below; 2 = Diploma; 3 = Bachelor; 4 = Master or above. Organization type: 1 = State-owned enterprise; 2 = Private enterprise; 3 = Foreign-owned enterprise; 4 = Public institutions; 5 = Other type. *p < 0.05, **p < 0.01.

### Common Method Variance Testing

The common method bias may have resulted because all variables were self-reported. Consistent with previous studies, we used Herman’s one-way test for testing and SPSS 25.0 for analysis (Podsakoff et al., 2012). The test results show that for factors with eigenvalues greater than 1, there are six factors with a cumulative variance contribution of 69.453%. The percentage of variance explained by the first common factor was 26.609%, which was below the critical value criterion of 40%. Thus, there is no serious common method bias in this study.

### Confirmatory Factor Analysis

To examine the discriminant validity between the main construct (felt accountability, obsessive passion, work overload, task performance, emotional exhaustion) and the corresponding measurement parameters of each scale, we performed confirmatory factor analyses (CFA) using AMOS 24.0. Specifically, according to Jackson et al. (2009), we compared the model fit between different models *via* fit indices such as CFI, TLI, RMSEA. The results indicated that the five-factor model provided a better fit to the data (χ^2^ = 733.309, df = 377, CFI = 0.914, TLI = 0.904, IFI = 0.915, RMSEA = 0.068), compared with other comparative models. Given the results, we suggest that the five constructs are empirically distinct.

### Hypothesis Testing

**FIGURE 2:**
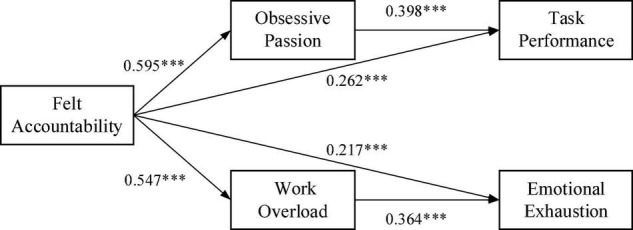
Results from the path analysis. To make the model diagram more concise, the path coefficients of the control variables to the dependent variable are not directly presented in the graph. ***p < 0.001.

We tested all hypotheses by conducting path analyses using AMOS 24.0. Hypotheses H1a and H1b proposed that felt accountability is positively related to both task performance and emotional exhaustion. In support of Hypotheses H1a and H1b, as shown in Figure 2 and Table 3, felt accountability significantly predicted task performance (β = 0.262, *p* < 0.001), and emotional exhaustion (β = 0.217, *p* < 0.001). Therefore, Hypotheses H1a and H1b were initially supported.

**TABLE 2:** Results of confirmatory factor analysis.

Model	*χ^2^*	df	*χ^2^/df*	RMSEA	IFI	TLI	CFI
Five-factor model	733.309	337	2.372	0.068	0.915	0.904	0.914
Four-factor model	1131.304	341	3.235	0.087	0.860	0.844	0.859
Three-factor model	1704.186	344	4.954	0.116	0.750	0.723	0.748
Two-factor model	2365.111	346	6.836	0.141	0.628	0.592	0.626
One-factor model	3260.322	347	9.396	0.169	0.464	0.412	0.461

*N = 294. One-factor model: Felt accountability + Obsessive passion + Work overload + Task performance + Emotional exhaustion. Two-factor model: Felt accountability + Obsessive passion + Work overload + Task performance, Emotional exhaustion. Three-factor model: Felt accountability + Obsessive passion + Work overload, Task performance, Emotional exhaustion. Four-factor model: Felt accountability + Obsessive passion, Work overload, Task performance, Emotional exhaustion. Five-factor model: Felt accountability, Obsessive passion, Work overload, Task performance, Emotional exhaustion. The symbol “+” indicates that the variables are combined.*

**TABLE 3:** Results of testing the hypotheses.

			Coeff	SE	CR	*P*
Felt accountability	→	Work overload	0.815	0.102	7.968	***
Felt accountability	→	Obsessive passion	0.442	0.086	5.158	***
Work overload	→	Emotion exhaustion	0.353	0.069	5.082	***
Obsessive passion	→	Task performance	0.533	0.119	4.474	***
Felt accountability	→	Emotion exhaustion	0.378	0.13	2.898	**
Felt accountability	→	Task performance	0.216	0.095	2.276	*

Unstandardized coefficients. SE, standard error. *p < 0.05, **p < 0.01, ***p < 0.001.

Hypotheses 2a and 2b proposed the mediation effects of obsessive passion between felt accountability and task performance. As shown in Figure 2 and Table 3, felt accountability was positively associated with obsessive passion (β = 0.595, *p* < 0.001), and obsessive passion was positively associated with task performance (β = 0.398, *p* < 0.001). Thus, Hypothesis 2a and H2b were supported. Hypothesis 3a and 3b proposed the mediation effects of work overload between felt accountability and emotional exhaustion. As shown in Figure 2 and Table 3, felt accountability was positively associated with work overload (β = 0.547, *p* < 0.001), and work overload was positively associated with emotional exhaustion (β = 0.363, *p* < 0.001). Thus, H3a and 3b were also initially supported.

Furthermore, to demonstrate more robust results, following the suggestion of Preacher et al. (2007), we tested the significance of the indirect effect of felt accountability on the two dependent variables (task performance and emotional exhaustion) through the two mediating variables (obsessive passion and work overload). Specifically, we calculate 95% confidence intervals for the estimated effects based on a random sample of 5000 bootstraps. If the confidence interval excludes zero, then the indirect effect is statistically significant. The results in Table 4 indicate that the indirect effect of felt accountability on task performance through obsessive passion was 0.237, with a 95% confidence interval of (0.127, 0.387). Similarly, the indirect effect of felt accountability on emotional exhaustion through work overload was –0.220, with a 95% confidence interval of (–0.347, –0.132). Taking these findings together, Hypotheses H2a, 2b, 3a, and 3b are adequately supported.

**TABLE 4:** Results of the mediating effects of obsessive passion and work overload.

Indirect effects *via*	*SE*	Effect	*P*-value	Bootstrapping 95% CI	
				LLCI	ULCI
Path 1 FA→WO→EE	0.053	0.199	0.001	0.107	0.318
Path 2 FA→OP→TP	0.064	0.237	0.001	0.127	0.387

*N = 294. FA, Felt accountability; OP, Obsessive passion; WO; Work overload; TP, Task performance; EE, Emotional exhaustion.*

## Discussion

### Theoretical Implications

First, we complement the literature on felt accountability by examining a double-edged sword effect of felt accountability. Previous studies have pointed out that felt accountability is a complex phenomenon, but it is not sufficiently understood. Almost all studies have examined the role of felt accountability based on a single pathway, focusing either on the “positive side” (Mero et al., 2014) or on the “dark side” (Hochwarter et al., 2005; Lanivich et al., 2010), ignoring the complexity of felt accountability. Hall et al. (2017) proposed that felt accountability may have different effects on work outcomes and individual wellbeing. For the first time, we provide empirical support for this view by placing job performance and individual wellbeing within a mode, examining the double-edged sword effect of felt accountability. The findings create a more nuanced understanding of the complex relationship between felt accountability, work outcomes, and individual wellbeing.

Second, by extending the controlled motivation perspective of self-determination theory into the domain of felt accountability, this study expands the application of self-determination theory. Even though, Ryan and Deci (2000a) proposed two types of motivation, autonomous and controlled motivation. However, previous studies have focused more on autonomy motivation and have neglected the use and exploration of the controlled motivation. Some studies contend that there is no such thing as free will because there are no situations where human behavior is entirely independent of external influences (Miles, 2012). Thus, autonomy motivation is hard to obtain and is fragile due to the demanding environmental requirements. Processes of controlled motivation generation, such as felt accountability, are more prevalent and should be given more attention. In the context of our study, we propose that felt accountability is a typical manifestation of individuals’ organic integration with external norms. Specifically, we conceptualize felt accountability as a controlled motivation, generate from the controlled internalization process. This perspective complements and enriches the component of controlled motivation, and contributes to the overall development of self-determination theory.

Third, we provide new and meaningful insight into the motivational process of accountability. Almost all studies are based on role theory or resource conservation theory, but neither of these perspectives provides a comprehensive explanation. Hall et al. (2017) proposed that motivation-related theories are concerned with the direction, level, and persistence of effort, which is related to the process of felt accountability. Resonating with this viewpoint, our study interprets felt accountability from the perspective of internalization and motivation, bridging the shortcomings of previous theories and providing a more comprehensive explanation for the double-edged sword effect. Specifically, we integrate self-determination theory and role theory to reveal why and how felt accountability can have different effects on task performance and emotional exhaustion by examining the mediating role of obsessive passion and work overload.

Finally, the results of our data analysis also revealed some interesting findings that suggest potential issues for future research. First, we conducted a supplementary analysis of two cross-cutting pathways despite the two pathways indicated in the theoretical hypothesis section. We found that felt accountability has a negative indirect relationship with task performance through work overload. Surprisingly, our results also show that felt accountability has a negative indirect relationship with emotional exhaustion through obsessive passion (β = –0.446, *p* < 0.001). At first glance, this finding seems confusing since previous studies have primarily examined the negative effects of obsessive passions. After further consideration, we believe that this finding is plausible, as it may be related to our sample, providing results different from those of developed countries. Due to the high competition brought about by rapid economic development, employees in China are generally held accountable for reasons such as strict performance evaluations. To avoid the harmful effects of frequent breakdowns, individuals need to self-regulate under intense work. Employees with obsessive passions put more energy into their work, buffering their uncertainty and anxiety about the future. Other research has similarly shown that obsessive passion may have mixed effects, reflecting its conflicted nature as compensatory striving to meet psychological needs (Lalande et al., 2017). For example, obsessive passion positively impacts organizational identification (Astakhova and Porter, 2015), positive affect, and flow (Forest et al., 2011; Pollack et al., 2020). Second, the results of our data analysis associated with the control also revealed some interesting findings that suggest potential issues for future research. As shown in Table 3, employee age was positively associated with felt accountability (γ = 0.154, *p* < 0.01) and obsessive passion (γ = 0.245, *p* < 0.01). A possible explanation could be that older employees who have risen to leadership positions need to take on more responsibilities.

### Practical Implications

The findings of this study also have practical implications for organizational management. To achieve improve employee execution and achieve organizational goals, managers often use the tool of accountability. The controlled motivation process associated with felt accountability is prevalent. These findings help managers understand the double-edged sword effect of felt accountability and provide practical suggestions for managing employee stress. First, there is nothing wrong with managers pursuing performance indicators. Still, the role of accountability should be viewed dialectically, as it not only brings benefits but also takes a toll on individual psychology. However, the mental health of employees is critical to the sustainability of the organization. Therefore, to avoid psychological damage, managers should give employees time and opportunity to recover after using accountability to achieve performance goals (Cheong et al., 2016). In addition, managers need to provide mental health resources to employees by creating a more open and inclusive culture.

Second, this study allows managers to think deeply about the real purpose of accountability, motivating employees to work proactively. Therefore, managers need to use accountability appropriately to achieve the desired results. For example, managers should set reasonable punishment norms and boundaries when implementing accountability, consider employees’ past attitudes and performance, and make appropriate allowances for non-principled failures.

Third, employees can deepen their understanding of the accountability system by understanding the double-edged sword effect of felt accountability, then learn to respond to the expectations of others in a positive way. Although employees often have to endure accountability pressures and force themselves to work, healthy emotion is key to long-term success. In other words, employees should identify objective responsibility and improve mental toughness and adaptability.

## Limitations and Future Research Directions

The data in the study are self-reported since the model we constructed largely reflects intrapsychics. Although no serious common method variance was tested, there may still be some impact. Our data were collected in three rounds, and some subjects were lost in each round of data collection. Although we evaluated for sample bias in our analysis and found no significant risk to the results, future studies could use more advanced designs that provide more conservative and robust support for the relationships between the proposed variables. Future research could use a multiple-source data approach to validate the causal relationships between variables by having leaders report individual task performance or having family members report individual levels of emotional exhaustion. Additionally, future research could also use empirical sampling methods to track the dynamics of felt accountability.

Second, our study focused on the different paths of felt accountability on individual wellbeing and work outcomes. Still, these relationships may differ under contingent conditions, and future studies could test the boundary conditions for different paths. These boundary conditions may be individual-level, such as a leader’s personality, values, and attributions, or team-level, such as their leadership style. For example, causal orientation, as a personality trait, describes an individual’s motivational orientation and is generally characterized by autonomy orientation, control orientation, and impersonal orientation. Individuals with high levels of autonomy orientation tend to take actions guided by their values interests, and the meaning of their work (Deci et al., 2017). Whether or not felt accountability make individuals feel in control, individuals with autonomous orientations are more likely to find meaning and pleasure in their work in the midst of stress. As a result, they experience more work passion rather than the psychological burden.

The third limitation is related to the measurement of felt accountability that we use. We used the scale of felt accountability for the first time in a Chinese context. Although it had good reliability, we did not revise the scale.

Unlike the individualism emphasized in western culture, China is a highly collectivist country, attaching more importance to the morality and obligations of individuals to the collective. In addition to meeting the expectations of others, employees in Chinese organizations may have a more complex understanding of accountability, which includes work ethic, dedication, and self-sacrifice. However, the items for the Hochwarter et al. (2007) mainly focus on evaluation expectations, felt accountability in the Chinese context may have overlooked the connotations of obligation and morality. Future research could consider the specificity of the context and develop a targeted scale, or conduct cross-cultural comparative studies to explore the effects of felt accountability in different cultural contexts.

## Conclusion

Felt accountability is demonstrated to have a complex effect on employee behavior and wellbeing. However, past research has ignored whether and how felt accountability has different effects on work outcomes and individual wellbeing. Relying on self-determination theory, we illustrate the double-edged effect of felt accountability. Our study shows that felt accountability had a significant positive effect on both emotional exhaustion and task performance. Specifically, felt accountability exacerbates emotional exhaustion by imposing work overload, while it induces obsessive passion, which in turn promotes employees’ task performance.

## Data Availability Statement

The raw data supporting the conclusions of this article will be made available by the authors, without undue reservation.

## Author Contributions

GY and YL designed the research, performed the research, analyzed the data, and wrote the manuscript. WL provided comments on different versions of the manuscript. All authors made substantial contributions to the manuscript and approved it for publication.

## Conflict of Interest

The authors declare that the research was conducted in the absence of any commercial or financial relationships that could be construed as a potential conflict of interest.

## Publisher’s Note

All claims expressed in this article are solely those of the authors and do not necessarily represent those of their affiliated organizations, or those of the publisher, the editors and the reviewers. Any product that may be evaluated in this article, or claim that may be made by its manufacturer, is not guaranteed or endorsed by the publisher.
